# Supplementary material for: Unlocking the Potential of Chemically Modified Carbon Gels in Gallic Acid Adsorption
Source: Gels. 2024 Feb 2;10(2):123. doi: 10.3390/gels10020123 (PMC10887651; doi:10.3390/gels10020123)
Supplement: Supplementary file 1 [file gels-10-00123-s001.zip › gels-2817735-supplementary.pdf]

EDX composition

Table S1. Elemental composition (EDX).

Sample	%C	%N	%S	%O
C-X	85.9	0.0	0.0	14.1
C-X-S	89.6	0.0	1.4	9.1
C-X-N	85.2	0.0	0.0	14.8
C-X-Na	88.8	0.0	0.0	11.3
C-X-K	90.5	0.0	0.0	9.5
C-X-Na + GA	90.5	0.0	0.0	9.3

In some samples small amounts of elements such as F or Cl have been found, so that the composition shown may not be 100 %.

FTIR band assignation table

Table S2. FT-IR spectra of the samples. Band assignment.

Wavenumber (cm^{-1})	Assignment[a]	Atomic grouping
3650-3200	ν(O-H)	Hydroxy group, H-bonded OH stretch
	ν(N-H)	Primary amine, NH stretch
3000-2800	ν(C-H)	Hydrocarbons (aliphatics, olefinic, and aromatic)
1770-1650	ν(C=O)	Carbonyl compounds
1700-1500	ν(C=C)	Olefinic and aromatic structures
1480-1400	δ(C-H)	Aliphatic structures
1450-1200	δ(C-H)	Olefinic and methyl groups
	δ(O-H)	Primary or secondary alcohol and carboxylic acid
1300-1200	ν(C-O)	Ether structures and phenol
1210-1100		Tertiary alcohol
1120-1070		Secondary alcohol
1075-1000		Primary alcohol
900-700	γ(C-H)	Aromatic structure

[a]Abbreviations: ν, stretching; δ, bending (in-plane); γ, bending (out-plane).

Kinetic models

The kinetic data were fitted to the pseudo-first-order (Lagergren), pseudo-second-order (Ho and McKay), and Weber and Morris kinetic models. The pseudo-first-order equation rearranged to a linear form is usually written as Equation (1):

$$log(q_e - q_t) = log\ q_e - \frac{K_1}{2.303}t \qquad (1)$$

Where q_e and q_t are the amounts of GA adsorbed at equilibrium and at time t (mg g^{-1}), respectively, and K_1 is the pseudo-first-order rate constant (h^{-1}). The representation of log(q_e - q_t) versus t should be a straight line with slope log q_e and intercept -K_1/2.303.

Assuming that the adsorption capacity is proportional to the number of active sites occupied on the surface of the adsorbent, the linear form for the pseudo-second-order reaction is written as equation (2):

$$\frac{t}{q_t} = \frac{1}{K_2 q_e^2} + \frac{1}{q_e}t \qquad (2)$$

Where q_e and q_t have the same meaning as in the previous model, and K_2 is the rate constant of pseudo-second-order adsorption (g mg^{-1} h^{-1}). Here, the plot of t/q_t versus t should generate a linear relationship, from which q_e and K_2 can be obtained from the slope and the intersection of the plot.

The model proposed by Weber and Morris allows us to know if the limiting step of the adsorption process is intraparticle diffusion. This model is not strictly kinetic but decides whether the adsorption rate depends on mass transfer within the adsorbent

particle or on other factors. The equation of this model in linear form is written as equation (3):

$$q_t = k_{id}t_e^{1/2} + C \qquad (3)$$

Where K_{id} is the intraparticle diffusion rate constant (mg g^{-1} $h^{-1/2}$), t_e is the equilibrium time (h^{-1}), and C is the intercept with the vertical axis (mg g^{-1}). This parameter gives an idea of the value of the boundary layer. The plot of q_t versus $t^{1/2}$ should provide a straight line in which K_{id} and C can be obtained from the slope and the intersection.

Isotherm models

The Langmuir and Freundlich isotherm models analyzed the adsorption of GA under equilibrium conditions. The isotherm model proposed by Langmuir admits that the solid has a limited adsorption capacity, where molecules are adsorbed on well-defined, energetically equivalent sites far enough away from each other not to allow interaction between molecules adsorbed on adjacent sites. This model assumes the following phenomena occur:

- Only one layer is covered over the surface. There is no multilayer.
- Each active site can only bind one adsorbate molecule.
- All active sites have the same adsorption energy.
- The ability to adsorb on an active site is independent of the occupancy of neighbouring sites.

Langmuir linear form is written as equation (4):

$$\frac{C_e}{q_e} = \frac{1}{S_m K_L} + \frac{C_e}{S_m} \qquad (4)$$

Where C_e is the equilibrium concentration in the liquid phase (mg L^{-1}), q_e is the amount adsorbed per unit of adsorbent mass (mg g^{-1}), S_m is the constant related to the maximum adsorption capacity (mg g^{-1}), and K_L is the Langmuir constant associated with the intensity of adsorption (L mg^{-1}). The representation of C_e/q_e against C_e generates a straight line; thus, from the slope and the intersection of the graph, $1/S_m$ and $1/S_mK_L$ can be obtained.

The Freundlich isotherm arises to explain adsorption in multilayers with interaction between adsorbed molecules. The application of this model suggests that the adsorption energy decreases exponentially once the adsorption active sites are complete. This model applies to adsorption on heterogeneous surfaces with a uniform energy distribution and reversible adsorption. The equation describing the isotherm is given in equation (5):

$$log\ q_e = log\ K_F + \frac{1}{n} log\ C_e \qquad (5)$$

Where q_e and C_e have the same meaning as in the previous model, K_F (mg g^{-1}) and 1/n are the Freundlich constants, with 1/n and K_F being computed from the slope and intercept of the Freundlich plot of log q_e against log C_e.

XPS spectra

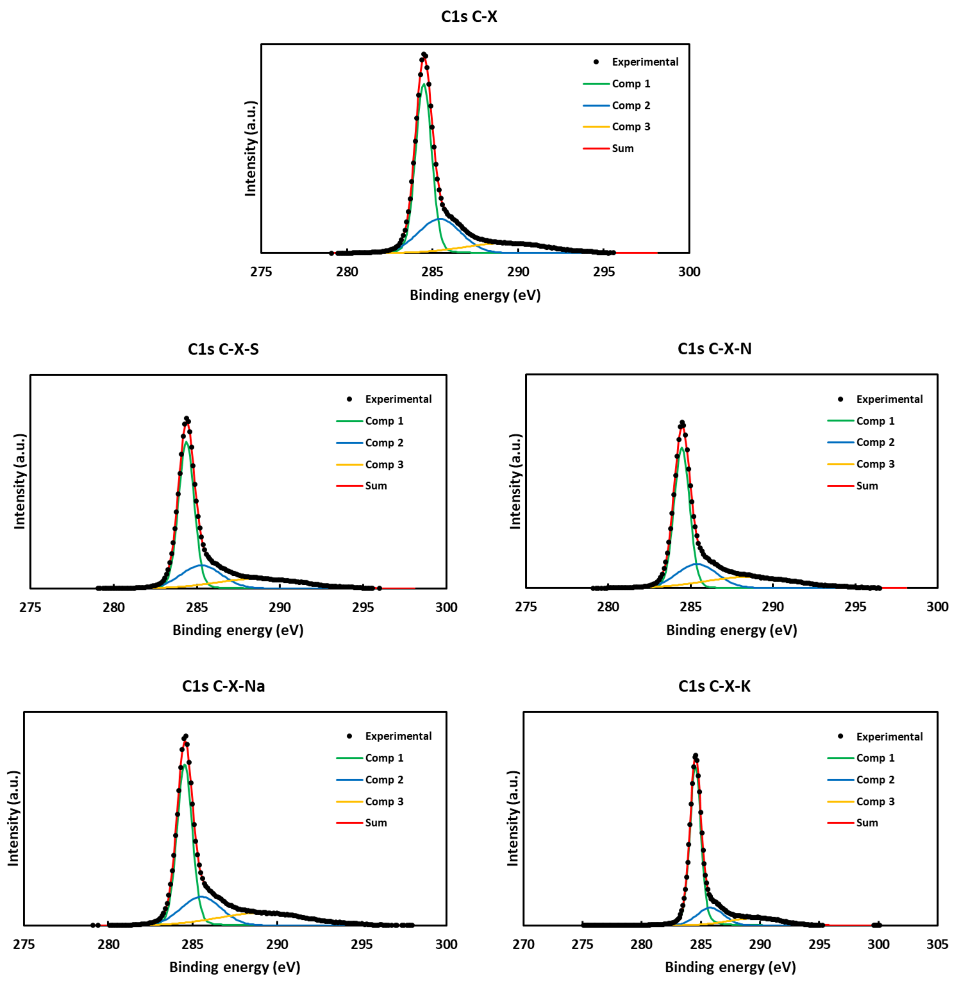

Figure S1. XPS spectra of C 1s.

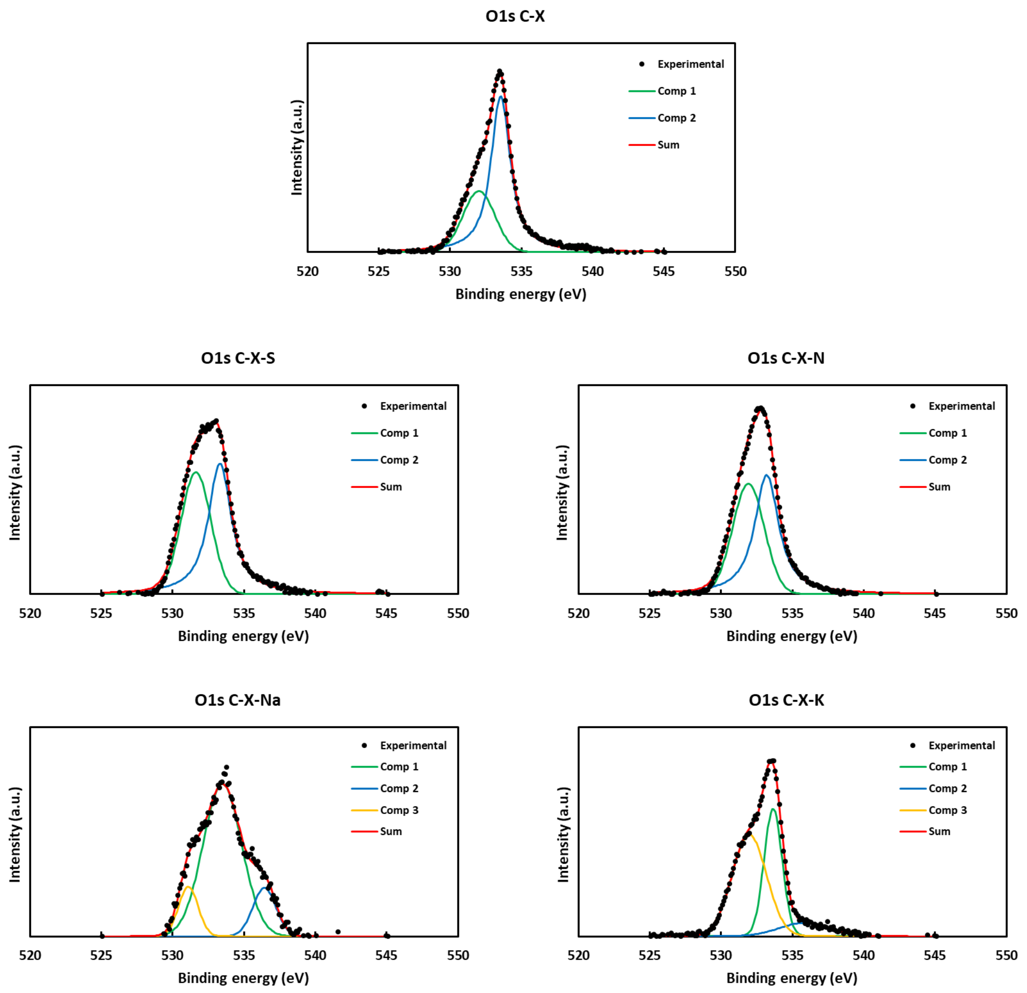

Figure S2. XPS spectra of O 1s.

SEM images

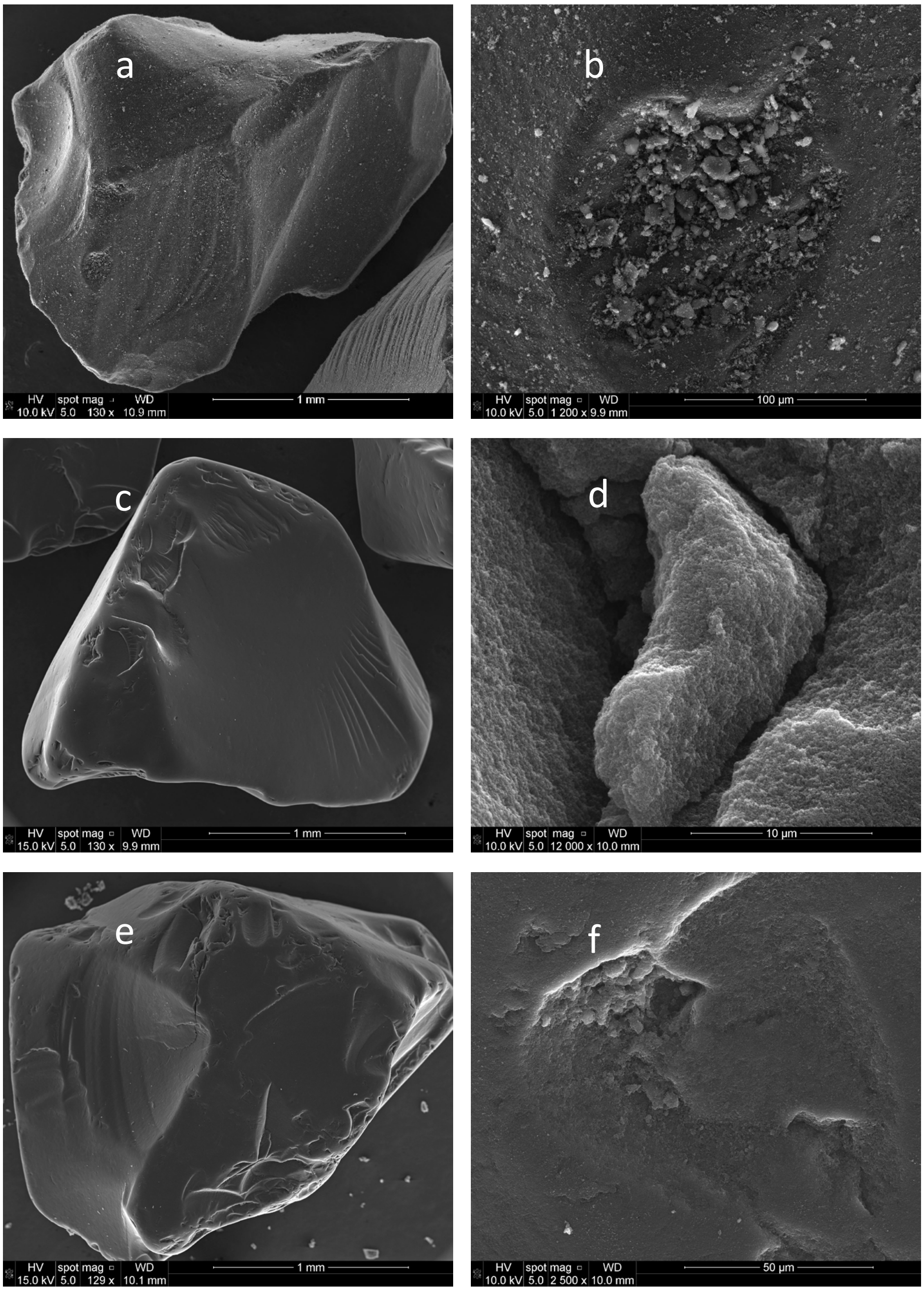

Figure S3. SEM images of C-X (a, b), C-X-N (c, d), C-X-S (e, f).

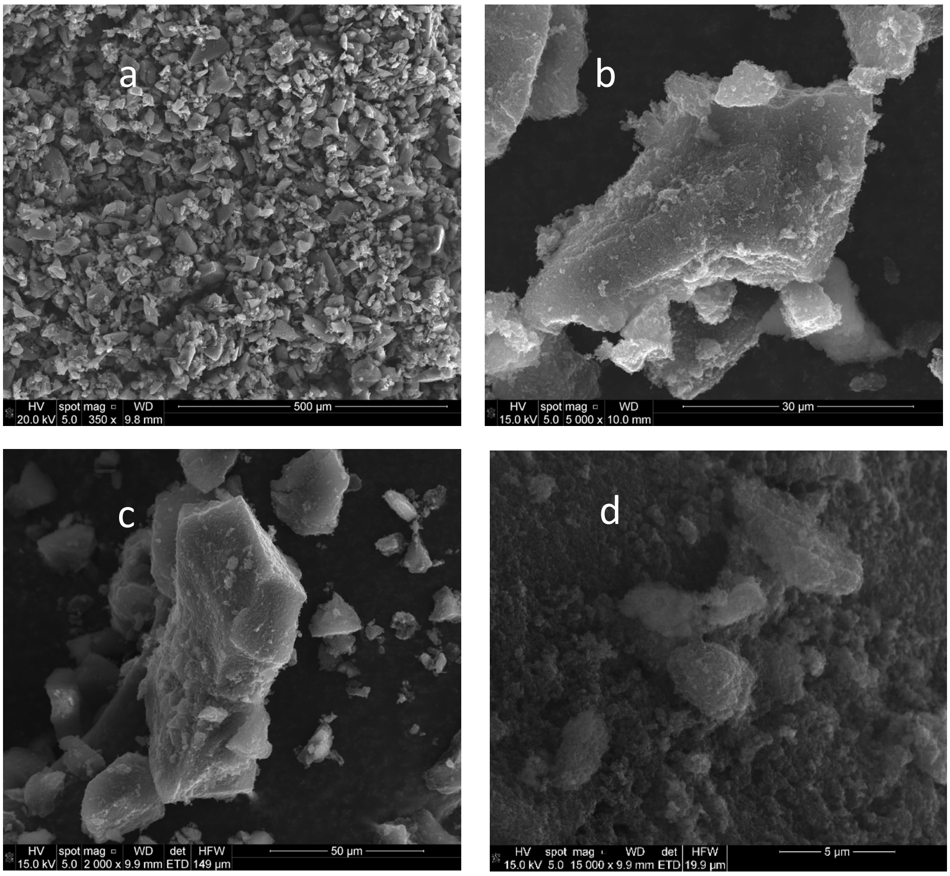

Figure S4. SEM images of C-X-Na (a, b), C-X-K (c, d).
